# Risk factors associated with *Acinetobacter baumannii *septicemia and its mortality rates in critically ill patients

**DOI:** 10.1186/cc12015

**Published:** 2013-03-19

**Authors:** K Kontopoulou, K Tsepanis, I Sgouropoulos, A Triantafyllidou, D Socratous, P Tassioudis, E Chasou, E Antypa, F Renta, E Antoniadou, K Mandraveli

**Affiliations:** 1 General Hospital G. Gennimatas, Thessaloniki, Greece

## Introduction

Acinetobacter baumannii (A. baum) is a leading cause of septicemia of patients hospitalized in the ICU with high mortality rates. The aim of our study is to investigate the risk factors associated with A. baum bacteremia and its mortality rates.

## Methods

A total of 937 patients (457 women and 480 men, median age 59) admitted to the ICU during the period 1 January 2009 to 30 September 2012 were enrolled in our retrospective study. Blood cultures were obtained from all patients. The identification and the antimicrobial susceptibility testing were performed by the automated system VITEK2 (Bio Merieux, France). Data collected included underlying diseases, malignancies and immune suppression (MIS), prognostic factors (APACHE score, adjusted mortality), age, sex, length of ICU stay (LS), recent administration of broad-spectrum ß-lactam antibiotics (especially carbapenems; ABL), mechanical ventilation (MV), implementation of invasive procedures (central venous catheter and urine catheter; INV.PR) and outcome. At first a univariate statistical model was used with significance level set at P = 0.05. For the multivariate statistical analysis model we used all variables with P <0.05 from the previous model and those mentioned at recent medical literature as significantly related with A. baum septicemia and its mortality.

## Results

A total of 101 patients (10.78%) developed bloodstream infection caused by A. baum and the mortality rate due to A. baum septicemia was estimated as 49.5% (50/101). Multiple regression analysis revealed adjusted mortality >55% (Exp(B) = 2.01, P = 0.013), MIS (Exp(B) = 1.97, P = 0.017), ABL (Exp(B) = 2.34, P = 0.009), LS >14 days (Exp(B) = 1.34, P = 0.034), MV (Exp(B) = 2.67, P = 0.005) and INV.PR (Exp(B) = 3.27, P = 0.001) as independent risk factors associated with A. baum septicemia.

## Conclusion

The fact that the probability of A. baum bacteremia increases in immunocompromised patients reffects the opportunistic characteristic of these infections. MV accelerates respiratory A. baum colonization, which is a risk factor for AB bacteremia. Recent INV. PR increased the incidence of A. baum bacteremia and this result is probably related to the severe status in patients with central venous catheter. The administration of carbapenems inhibits the growth of other more susceptible bacteria, allowing the growth of multidrug-resistant A. baum.
